# The hidden value of MRI: modifying treatment decisions in C-spine injuries

**DOI:** 10.1186/s13049-024-01235-9

**Published:** 2024-07-22

**Authors:** Niklas Rutsch, Florian Schmaranzer, Pascale Amrein, Martin Müller, Christoph E. Albers, Sebastian F. Bigdon

**Affiliations:** 1 Department of Orthopedic Surgery and Traumatology, Inselspital, Bern University Hospital, Freiburgstrasse, 3010 Bern, Switzerland; 2 Department of Diagnostic, Interventional, and Pediatric Radiology, Inselspital, Bern University Hospital, Freiburgstrasse, 3010 Bern, Switzerland; 3 Department of Emergency Medicine, Inselspital, Bern University Hospital, Freiburgstrasse, 3010 Bern, Switzerland

**Keywords:** Cervical vertebrae, Spinal injuries, Magnetic resonance imaging, Computed tomography, Neck injuries, Patient care management

## Abstract

**Background data:**

Computed Tomography (CT) is the gold standard for cervical spine (c-spine) evaluation. Magnetic resonance imaging (MRI) emerges due to its increasing availability and the lack of radiation exposure. However, MRI is costly and time-consuming, questioning its role in the emergency department (ED). This study investigates the added the value of an additional MRI for patients presenting with a c-spine injury in the ED.

**Methods:**

We conducted a retrospective monocenter cohort study that included all patients with neck trauma presenting in the ED, who received imaging based on the NEXUS criteria. Spine surgeons performed a full-case review to classify each case into “c-spine injured” and “c-spine uninjured”. Injuries were classified according to the AO Spine classification. We assessed patients with a c-spine injury detected by CT, who received a subsequent MRI. In this subset, injuries were classified separately in both imaging modalities. We monitored the treatment changes after the additional MRI to evaluate characteristics of this cohort and the impact of the AO Spine Neurology/Modifier modifiers.

**Results:**

We identified 4496 subjects, 2321 were eligible for inclusion and 186 were diagnosed with c-spine injuries in the retrospective case review. Fifty-six patients with a c-spine injury initially identified through CT received an additional MRI. The additional MRI significantly extended (geometric mean ratio 1.32, *p* < 0.001) the duration of the patients’ stay in the ED. Of this cohort, 25% had a change in treatment strategy and among the patients with neurological symptoms (AON ≥ 1), 45.8% experienced a change in treatment. Patients that were N-positive, had a 12.4 (95% CI 2.7–90.7, *p* < 0.01) times higher odds of a treatment change after an additional MRI than neurologically intact patients.

**Conclusion and relevance:**

Our study suggests that patients with a c-spine injury and neurological symptoms benefit from an additional MRI. In neurologically intact patients, an additional MRI retains value only when carefully evaluated on a case-by-case basis.

## Introduction

Cervical-spine (c-spine) injuries are common  [1, 2]. The c-spine is especially endangered in blunt acceleration/deceleration traumas. The typical trauma mechanisms include high-energy vehicle accidents and sports injuries, especially in younger patients  [3, 4], as well as low-energy traumas in elderly patients  [5].

Multi-detector computed tomography (CT) has evolved as the gold standard for c-spine evaluation and clearance  [6–14], as other modalities like X-Ray and LODOX^®^-Statscan do not share a sufficient sensitivity  [11, 14–25]. Recent studies challenge the superiority of the CT for the evaluation of c-spine trauma patients, as the sensitivity of CT is not sufficient to exclusively rule out an injury and stress the diagnostic value of magnetic resonance imaging (MRI). This is especially true for neurologically symptomatic patients with a negative CT scan  [25, 26].

However, the MRI is a costly, time-consuming imaging modality and not universally available  [27]. Simultaneously, in neurologically intact patients, MRI has been shown neither beneficial nor cost-effective for an adequate c-spine evaluation  [28]. Moreover, studies already observe overuse  [29], finally culminating in the use of cervical MRI for inappropriate indications  [30]. From a socioeconomic standpoint, in neurologically intact patients, a spine MRI poses a very costly modality for insurance companies and insured patients  [31].

In addition to costs, trauma situations demand rapid and correct treatment (ATLS Principle: Time is essential and do no further harm)  [32]. Patients who would benefit from surgery should receive it promptly  [33]. In this challenging context, the involved doctors always face the question of the additional benefit of further diagnostics. An MRI is not immediately available everywhere, and the examination also takes time. The time of treatment also plays a role in the overall efficiency of the emergency department (ED). With increasing numbers of patients in EDs, individual treatment should be as accurate, evidence-based, and efficient as possible. Unnecessary examinations should be avoided to conserve resources and capacities. Therefore, the question remains: is there a need for an additional MRI?

This retrospective mono-center study aims to understand the benefits and caveats of performing an additional MRI in injured c-spine trauma patients. Our goal is to identify patients that could benefit from an additional MRI which may improve patient outcomes and reduce the socioeconomic burden of excessive MRI use.

## Methods

This research draws upon a cohort previously studied by Rutsch et al. [25] with a different focus. While our previous research concentrated on sensitivity and specificity of different modalities in general, the current study takes a different angle by investigating the utility and impact of additional MRI on patients with CT-confirmed cervical spine injuries. The method and manuscript were prepared according to the STROBE guidelines [34].

### Study design and setting

We conducted a mono-center retrospective analysis of prospectively collected data, over ten years. All patients were admitted and treated at the ED, at the Inselspital, Bern University Hospital, Switzerland, which is the largest level-one trauma center and tertiary reference center in Switzerland.

### Eligibility

We identified all patients with trauma and neck pain between 01.01.2012 and 31.12.2017 that presented in the ED of Inselspital, University Hospital Bern (Switzerland). Of this population, we included all patients that required a radiographic c-spine evaluation according to the NEXUS criteria. We excluded all patients that presented at the pediatric ED and cases with incomplete medical records.

### Search strategy and data extraction

We searched the “ecare” (electronic patient records) database of the ED of Inselspital, University Hospital Bern (Switzerland), for the terms “HWS-Fraktur + Distorsion + Schleudertrauma + Whiplash + Halswirbel” (translation “HWS-fracture + distorsion + whiplash (german) + Whiplash + cervical vertebrae”), also including partial matches. We examined all the radiological examinations to classify the results as either "injured" or "uninjured," based on the reports given by the ED radiology staff at the time of imaging in the emergency room. Then, we carefully reviewed the patient records and images, and both a junior and a senior spine surgeon independently evaluated each patient to determine a final reference standard for “injury” or “no injury”. We resolved any differences in judgments through a multidisciplinary discussion. This cooperative approach ensured a thorough and clear evaluation of each case. To investigate the added value of an additional MRI we then focused only on patients with a positive CT report in the ED that received an additional MRI scan. We reviewed their imaging results and therapy in detail to classify the injury following AO Spine classification systems [35, 36]. The AO spine classification system allows a robust description of injury characteristics and severity of the upper cervical and sub-axial spine. Using the modifier variables, additional information, especially the presence of neurologic symptoms may be encoded [35, 36]. Then, we assessed the treatment strategy before and after additional MRI imaging was performed. The treatment strategy was assessed by a specialized spine surgeon who was unaware of the final treatment strategy and MRI imaging results. This way, using all available records and CT imaging results *prior* to the MRI, we identified the indented treatment strategy by the treating physician team in the patient records. If the treatment plan was not available, we imposed the standard treatment based on our best knowledge and practice as a level-1 spine trauma center. The data was extracted from the selected case files and transferred to an Excel spreadsheet. After anonymization, the data was imported into RStudio. The observational period concluded on December 31st, 2018.

### Variables

We extracted variables on patient demographics, patient symptoms, trauma mechanism, imaging, radiologic findings, and clinical management (e.g., performed surgery, vertebral level of surgery, anterior/posterior/combined surgical approach).

We set the time spend in the ED as a result of the time of admission until dismissal or transfer to a different hospital unit (i.e., intensive care unit, operation theatre). Every c-spine injury was classified utilizing the AO Spine classification system for the upper cervical and subaxial spine  [35–37] depending on the localization and including neurological evaluation and modifiers as proposed by the classification system. During the full case review of the c-spine injured patients with CT and additional MRI, the reviewing surgeons assessed the treatment strategy (surgical/non-surgical treatment, surgical approach [i.e., anterior/posterior/combined], level of surgery [level and number of involved vertebrae]) by the CT result and the MRI result separately.

### Statistical methods

The normal distribution of a variable was assessed visually using qq-plots and using the Shapiro–Wilk test. Demographic differences between the cohort that received CT and additional MRI and the remaining population were assessed using a two-sided *t*-test. The difference in time spent in the ED (between the three groups CT only, MRI only, CT & MRI) was assessed by the Kruskal–Wallis test followed by pairwise comparison with Wilcoxon rank sum test and corrected for multiple testing using the Holm method. Length of stay (LOS) was log-transformed to assess the geometric mean ratio (GMR). Using the log values of LOS, we created a log-level linear regression model of the LOS dependent on the imaging the patients received. We then transferred it into the equivalent exponential function. To infer the predictive value of the AO Spine Neurology and AO Spine Modifier variable in predicting a treatment change, we used a binomial logistic regression model. To simplify the prediction model, we binned, the AO N categorical variable into only a binary output, N0 and N-positive, consisting of all N1 and greater. The AO modifier variable consists of four distinct categories. Statistical analysis was performed using RStudio [38, 39] and the R-package “tidyverse” [40]. Data visualization was conducted using the R-package “ggplot2” [41] and Affinity Designer^®^ (Version 1.10.6, Serif (Europe) Ltd. The Software Centre, Wilford Industrial Estate, Nottingham).

## Results

Our database search strategy identified 4,996 patients of which 2,321 patients were eligible for inclusion. Our retrospective case review identified a total of 186 patients with a c-spine injury [25]. Out of the included patient population, 56 patients were diagnosed as injured in the initial CT and received a subsequent additional MRI during their stay at the ED.

### Demographics

CT-positive patients receiving an additional MRI, in comparison to the remainder of the cohort, were significantly more likely to be male (71.4% vs. 57.1%, *p* = 0.045), older (mean age: 58.57 years vs. 51.35 years, *p* = 0.014), and to have experienced high-energy trauma (44.6% vs. 26.3%, *p* = 0.004, Table 1).

**Table 1:** Demographics of the cohort that received an additional MRI compared to the remaining population

Demographic	Remaining population (n = 2265)		CT and additional MRI cohort (n = 56)		*p* value
Sex					0.045
Male	1294	(57.1%)	40	(71.4%)	
Female	971	(42.9%)	16	(28.6%)	
Age (years, mean)	51.3	(SD 22.8, range 15–101)	58.6	(SD 21.1, range 19–95)	0.014
Male	49.8	(SD 21.3, range 15–99)	56.2	(SD 20.7, range 19–95)	
Female	53.4	(SD 24.5, range 16–101)	64.4	(SD 21.5, range 20–89)	
Trauma mechanism					0.004
High energy	596	(26.3%)	25	(44.6%)	
Low energy	1669	(73.7%)	31	(55.4%)	
Nationality					
Swiss	1765	(77.9%)	43	(76.8%)	
German	66	(2.9%)	5	(8.9%)	
Other	434	(19.2%)	8	(14.3%)	

### Time in the emergency department

**Fig. 1:**
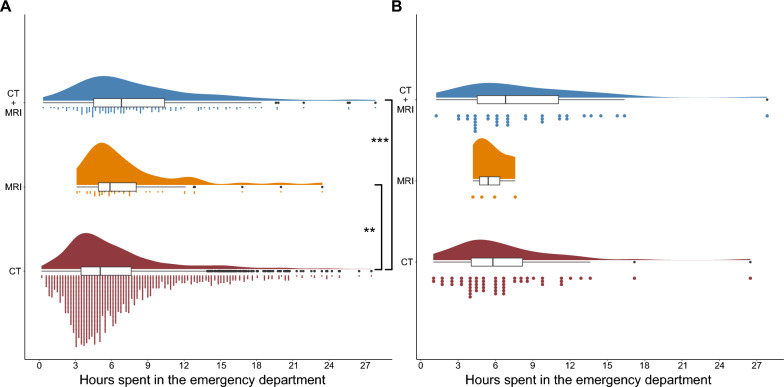
**A** Time spend in the emergency department of all patients who received CT, MRI, or in combination. Patients that receive both CT and MRI had a significantly longer LOS in the ED than patients that just received CT imaging (CT: 5.1 h [IQR 4.25]; CT and MRI: 6.8 h [IQR 5.9] *p* < 0.001***). Patients that received only an MRI stayed 1.28 times longer than the CT group (GMR 1.28, 95% CI 1.06–1.57, *p* = 0.011**). **B** Shows the LOS in the ED by the subset of patients, who resulted in injured in this cohort. There is no significant difference in time spent in the emergency department between the groups of patients classified as injured

In the complete patient population (n = 2,321), all patients with blunt neck trauma who received an additional MRI to a CT scan in the ED had a significantly longer length of stay (LOS) in the ED (CT: 5.1 h [IQR 4.25]; CT and MRI: 6.8 h [IQR 5.9] *p* < 0.001, Fig. 1A). The geometric mean of the LOS in the CT + MRI group was 1.32 times higher than in the CT group (GMR 1.32, 95% CI 1.18–1.48, *p* < 0.001). This resulted in the following linear regression model: $LOS=1.32*x+5.1$ (95% CI of coefficient 1.18–1.48, *p* < 0.001; 95% CI of Intercept 4.93–5.27, *p* < 0.001). Patients that received only an MRI stayed 1.28 times longer than the CT group (GMR 1.28, 95% CI 1.06–1.57, *p* = 0.011). Regarding only the group of patients who then were diagnosed with c-spine injury (n = 186), there was no significant difference in time spent in the ED (Fig. 1B).

### Diagnosis of injured patients who received MRI after a positive CT scan

**Fig. 2:**
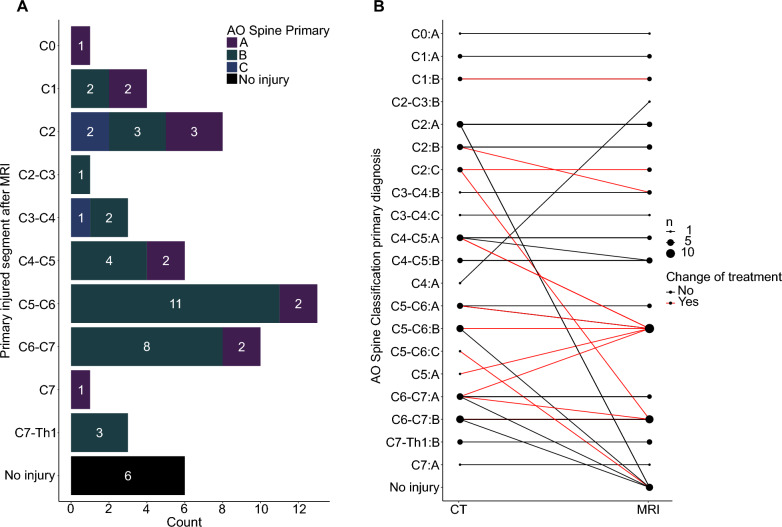
**A** Bar graph representing patients' retrospective primary AO diagnosis after case review with pathological CT finding and additional MRI. **B** The network shows the diagnosis and treatment change (red = change of treatment) from initial CT to the diagnosis and treatment decision after MRI results. The dot size corresponds to the number of patients with each injury, as classified by the AO spine system

After case review, the injury severities were encoded following the AO Spine injury classification systems  [35–37] as follows: A: 13 patients, B: 34 patients, C: 3 patients. The most frequently injured segment was C5–C6 with a type B injury (Fig. 2A). Six patients were classified as uninjured after the MRI (false-positive CT).

### Therapeutic impact of an additional MRI scan

The majority of patients (39 patients, 69.6%) had no change of classification, according to the AO Spine injury classification systems. Most frequently altered classifications were (n = 3, C4–C5 A; n = 3, C6–C7 A; Fig. 2B).

**Fig. 3:**
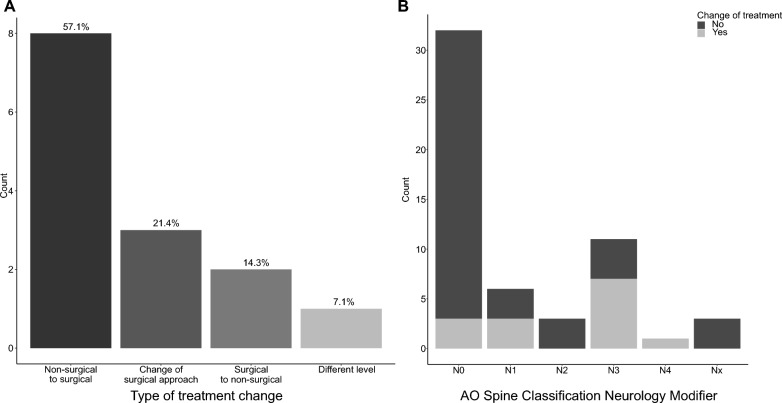
**A** Bar graph showing the number of patients with treatment change after the additional MRI in more detail. Most frequently, treatment change meant a change in strategy either from conservative to surgery or otherwise. **B** Bar graph representing the number of patients for each AO N (0–4, x) as classified in the ED. Patients with a treatment change after consecutive MRI are highlighted in grey

After the additional MRI, 14 patients (25%) had a change of treatment. Of those patients, nine (64.3%) had a change in their AO primary classification diagnosis after MRI (Fig. 2B). Most frequently, the indicative treatment change was related to determining a surgical treatment vs. a non-surgical treatment. In most of the patients with a treatment change (57.1%, n = 8), MRI uncovered additional findings (i.e., critical disc-herniation, extended myelopathy with ongoing bony compression) that changed the course of treatment to surgery (Fig. 3A). In 21.4% (n = 3), the treatment decision was changed from surgical to non-surgical. Moreover, MRI affected the type of surgical approach (anterior vs. posterior approach) used (14.3%, n = 2), and in one case altered the segment level of the surgery (7.1%, n = 1) (Fig. 3A).

### Patients with neurologic symptoms

In patients with intact neurology at the time point of a CT (n = 32), treatment changed after the additional MRI in 9.4% (n = 3). In patients with an AO N ≥ N1 (n = 24), in 45.8% (n = 11), the decision of treatment changed after MRI (Fig. 3B).

We conducted a logistic regression analysis to evaluate the effect of the AO neurology and modifier variable on treatment change after an additional MRI. Patients categorized as N-positive (N1–4, x) had 12.4 times higher odds (95% CI 2.70–90.65, *p* < 0.01) of a treatment change after the additional MRI than patients categorized N0.

### The value of the AO spine classification modifier

In the same model, we did not observe any significant predictive effect of the M-modifier of the AO spine classification system on predicting a treatment change after the additional MRI.

## Discussion

The value of an additional MRI in patients already diagnosed with a cervical spine (c-spine) injury remains a subject of controversy, given the varied clinical pathways and management approaches in play. Our study offers essential insights into this debate by exploring the clinical implications of supplemental MRI in assessing patients with CT-diagnosed c-spine injuries. The results underscore the diagnostic utility of this additional imaging modality in informing treatment alterations, particularly for patients presenting with neurological symptoms. However, the extended duration of ED stays associated with this strategy merits consideration. Consequently, our findings add a nuanced perspective to this ongoing discourse and could guide efforts toward optimized resource allocation and improved patient care outcomes.

### MRI prolongs the diagnostic process

This study set out to examine the diagnostic benefit of an additional MRI in this specific cohort of patients with blunt c-spine trauma and an initial pathologic CT finding. Generally, we demonstrate, that patients with an additional MRI (no matter the final diagnosis) spend a substantially longer time in the ED. This relates to the additional time required to coordinate and obtain an MRI. The CT acts as the gold standard to rule out a c-spine injury and leads to a median stay of around five hours in the ED. When an additional MRI was performed, the time of stay in the ED was significantly increased by more than one hour, potentially binding critical resources in the ED and producing high additional costs for the health care system  [27]. Apart from the socioeconomic impact, the additional MRI also significantly prologues the time spend in a cervical collar  [27].

### The diagnostic and therapeutic value of an additional MRI

Even if CT is not perfectly accurate, it remains the gold standard for diagnosing cervical spine injuries due to its universal availability and fast result. However, practitioners should be aware that there is a significant proportion of missed injuries on CT, as we previously reported a sensitivity of 88.6%  [25]. Thus, we warrant further prospective studies investigate an MRI's benefits in patients with negative CT  [13, 25, 26]. In selected patients, especially with neurologic symptoms, this is where the MRI holds a strong value  [25] revealing undetected injuries. However, our study took a different approach. This study questions the medical justification of additional cervical MRIs after already having diagnosed a c-spine injury in the initial CT. Here, we aim to decipher, which patients with an already positive CT finding benefit from an additional MRI to prevent over-diagnostics and provide a more cost-effective diagnostic process in the future.

**Fig. 4:**
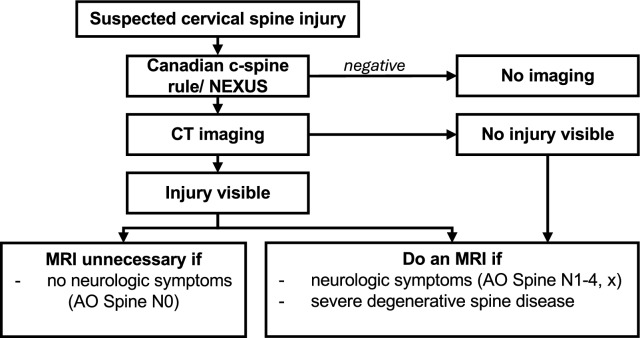
This presents our proposed clinical algorithm for imaging diagnostics of cervical spine injuries. Following a thorough clinical examination, the decision for c-spine imaging is determined by the Canadian c-spine rule and/or NEXUS criteria. CT imaging is established as the gold standard modality for detecting c-spine injuries. An additional MRI should be considered if the patient exhibits neurological deficits or severe degenerative spine disease. Based on our findings, this is also the case if a c-spine injury is already identified on the CT scan

We demonstrate that MRI has rarely significantly affected patient care if an injury had already been diagnosed. Especially when patients were neurologically asymptomatic, we did not observe a change of treatment course in 90.6% of the patients. In these neurologically intact patients, treatment only changed twice from non-surgical to surgical (underestimation of injury in CT), and once from surgical to non-surgical. Thus, in these patients, an MRI should only be used after careful consideration of the individual patient to retain added value (Fig. 4).

Patients with diagnosed injury and any neurologic deficit were significantly more likely to benefit from the additional MRI. It either changed the surgical approach (anterior/posterior/level) or the treatment strategy (surgical vs. non-surgical). Following our in-house standards, if the patient suffered from a mono-level injury or critical disc herniation, we usually approach from the anterior. In the case of multilevel stenosis with myelopathy, we favor the approach from the posterior.

Therefore, we emphasize the benefits of an additional MRI in patients with a diagnosed c-spine injury by CT and the presence of neurologic symptoms (Fig. 4). We urge for the distribution and correct implementation of the AO Spine classification, as we highlighted the diagnostic and therapeutic impact of the AO Spine Neurology modifier.

### Strengths

This extensive cohort study, based on a substantial duration and substantial patient pool, elucidates the consequences of additional MRI usage in a real-world, Level-I trauma setting. With 2321 cases analyzed, our research provides crucial insights into a specific, yet contentious, patient subgroup with CT-detected c-spine injuries who also undergo an MRI. Our investigation pioneers in exploring not only the diagnostic influence of an extra MRI but also its effect on treatment trajectory in an ED setting using the standardized AO Spine classification [35, 36] system.

### Limitations

As previous results from this patient cohort have already been published  [25], this study may perpetuate any potential bias as it represents the same population based on a single trauma center. This bias may be mitigated as this study results from a very distinct subgroup analysis of an initially large sample size and by its real-world setting. However, to really identify the patient subpopulation that will most likely benefit from routine additional MRI imaging, this study is limited by its retrospective observational design. We warrant prospective studies for further investigations. In terms of imaging methods, different CT and MRI imaging protocols were included. Moreover, not all patients with a positive CT finding received an additional MRI, thus, leaving the potential for the introduction of sampling bias.

## Conclusion

Our study provides a critical evaluation of the implications of additional MRI use in the diagnosis of c-spine injuries. We have demonstrated that an adjunctive MRI extends patients' ED stays, increasing resource allocation and potentially affecting cost-effectiveness. However, our findings highlight that this change in diagnostic protocol only significantly impacts a subset of patients: those with neurological symptoms. For these patients, an additional MRI is more likely to lead to a treatment alteration. Conversely, in patients without neurological impairment, an additional MRI appears less critical, suggesting opportunities for more selective and cost-effective diagnostic strategies. Our research underscores the importance of tailored diagnostic approaches in managing c-spine injuries, furthering the ongoing discourse surrounding optimal resource utilization and patient management in trauma settings.

## Data Availability

The datasets used and/or analyzed during the current study are available from the corresponding author on reasonable request.

## Abbreviations

c-spine: Cervical spine
CT: Computed tomography
ED: Emergency department
GMR: Geometric mean ratio
HRA: (Swiss) human rights act
LOS: Length of stay
MRI: Magnetic resonance imaging
